# Green Strategies of Powdery Mildew Control in Hop: From Organic Products to Nanoscale Carriers

**DOI:** 10.3390/jof7060490

**Published:** 2021-06-19

**Authors:** Alejandra J. Porteous-Álvarez, M. Mercedes Maldonado-González, Sara Mayo-Prieto, Alicia Lorenzana, Ana I. Paniagua-García, Pedro A. Casquero

**Affiliations:** 1 Grupo Universitario de Investigación en Ingeniería y Agricultura Sostenible (GUIIAS), Escuela de Ingeniería Agraria y Forestal, Universidad de León, 24009 León, Spain; apora@unileon.es (A.J.P.-Á.); mmalg@unileon.es (M.M.M.-G.); smayp@unileon.es (S.M.-P.); alorv@unileon.es (A.L.); 2 Centro de Biocombustibles y Bioproductos, ITACyL—Instituto Tecnológico Agrario de Castilla y León, Villarejo de Órbigo, 24358 León, Spain; pangaran@itacyl.es

**Keywords:** *Humulus lupulus*, *Podosphaera macularis*, yield index, α-acids yield, nanoscale carriers, fungicides, Nugget cultivar, integrated disease management

## Abstract

*Humulus lupulus* L. is a long-lived, perennial, herbaceous, and dioecious climbing plant. The foremost producers in the European Union are Germany, the Czech Republic, Poland, Slovenia, and Spain. The Spanish cultivated area is concentrated in the province of León. Powdery mildew, caused by *Podosphaera macularis,* menaces hop production and quality in all hop growing regions located in the Northern hemisphere, colonizing leaves, petioles, inflorescences, and finally cones. In this work, powdery mildew control was monitored, comparing nine fungicide strategies: five organics, two integrated disease management (IDM)-based, with and without Nutragreen^®^ nanoscale carrier, and two conventional treatments (CON) with and without Nutragreen^®^ nanoscale carrier. The organic treatments were able to diminish *P. macularis* on leaves, but no effect was observed in cones. CON treatments reduced the infection on leaves and cones and increased the cone quantity and quality. Likewise, IDM-based treatments provided satisfactory results as they diminished powdery mildew on leaves and cones. Finally, dose reduction using a Nutragreen^®^ nanoscale carrier showed beneficial effects in the control of powdery mildew compared to the commercial dose. Hence, the use of nanoscale carries permits a 30% reduction in pesticide dose, which optimizes yield and hop quality, reduces risks linked to pesticides, and aids in compliance with public and international policy demands.

## 1. Introduction

*Humulus lupulus* L. is a long-lived, perennial, herbaceous, and dioecious climbing plant. Although it is cultivated worldwide, hops are mostly circumscribed to a narrow range of latitudes in temperate regions (moderate temperature and rainfall), between 35° and 55° latitude, both in the Northern and Southern hemispheres [1].

One of the principal diseases affecting hop crops is powdery mildew. This pathology is caused by *Podosphaera macularis* (Wallr.) U. Braun and S. Takam (formerly *Sphaerotheca macularis* (Wallr.) W.B. Cooke), wich menaces hop production and quality in all hop growing regions located in the Northern hemisphere [2]. This fungus colonizes, along the hop season, leaves, petioles, inflorescences, and finally cones. Infection of leaves infrequently produces economic losses but contributes to a source of inoculum for inflorescences and cones prompting their abortion, malformation, and discoloring, thus reducing yield and quality [3,4] and affecting the economic balance with the usage of phytosanitary products [5]. However, the potential effects of powdery mildew on both yield quantity and quality are still poorly understood [2,6].

Some authors associate premature ripening of cones with *P. macularis* infections on bracts and bracteoles located inside the cone, considerably hindering their observation [7,8]. However, it is still unknown how the colonization of this fungus can alter yield, quality, and beer features. Some authors stated that when 50% or less of the cones are affected by powdery mildew, no defects in the aroma and flavor were detected [9]. Likewise, another previous study showed that values of the incidence of powdery mildew of 17 to 23% did not produce alterations in cone yield or α-acid content of cones [10].

Reliable control of powdery mildew in the hop is problematic since its causal agent, *P. macularis*, can persist across winter as asexual mycelia or sexually derived cleistothecia within and on buds [11,12]. Hence, the severity and intensity of the disease caused by *P. macularis* at the beginning of each season are unknown. Hop farmers have to face the uncertainty of reducing the inoculum in leaves to decrease *P. macularis* colonization in cones. Some fungicides vary in their efficiency for controlling hop powdery mildew on leaves and cones. Their ability to manage powdery mildew on leaves was not necessarily prognostic of an accurate efficacy on cones, which leads to the necessity of evaluating both tissues in efficiency trials [13]. On the other hand, some studies have focused on controlling the powdery mildew by removing the basal leaves [4,14] or increasing plant resistance [2]. However, the knowledge about the direct effect of powdery mildew on both quantity (yield index) and quality (α-acid content) of cones is scarce [2,6,7].

Thus, a better knowledge of how *P. macularis* affects yield and quality of cones is crucial to building a robust disease management strategy, which must rely on sustainable intensification of crop production while reducing risks linked to pesticides, and following public and international policy demands.

The number of fungicides permitted in the control of powdery mildew has been reduced due to the 2013 Reform of the Community Agricultural Policy of the European Union, by which a new, greener approach to agriculture was established and a continuous reduction in the number of authorized synthetic pesticides was ushered in, which has led to giving priority to the non-chemical methods described in integrated production.

In this work, we evaluated the performance of eight greener alternatives and compared them with the conventional treatment in the control of powdery mildew in hops.

Nanotechnology is a new technology that has proven to have good performance in diverse fields within biology due to good biocompatibility [15,16,17] that has led to new applications in precision biology [18]. The application of this technology in agriculture may reduce the environmental impact of pesticide usage due to a better delivery of the products within the plant [14,19]. In pest management, Zheng et al. [20] developed a nanoscale polyamino acid to efficiently deliver insecticidal proteins to kill resistant pests. Carro-Huerga et al. [21] proved that the use of Nutragreen^®^ was able to control the disease in a pear orchard by efficiently delivering a reduced pesticide dose through the plant.

The main objective of the present study was to control powdery mildew in hops while also reducing the risks linked to pesticides by different means, either by evaluating various organic compounds or by evaluating different strategies based on integrated disease management with and without the use of a nanocarrier. The parameters observed consisted of the ability of field treatments to (i) reduce colonization of *P. macularis* in leaves; (ii) reduce *P. macularis* colonization in cones; (iii) maintain/increase cone yield; and (iv) maintain/increase the production of α-acids.

## 2. Materials and Methods

### 2.1. Field Sites, Treatments and Experimental Design

Two field experiments were conducted during 2020 in two hop yards planted with ‘Nugget’ cultivar located in León province (Spain), one in a commercial hop yard in ‘La Milla del Río’ (MR) (42°34’32.4″ N 5°51’19.1″ W) and the other in an experimental field of 0.72 ha of extension consisting of 40 rows situated in the School of Agrarian and Forest Engineering at the University of León (SAFE-ULE) (42°35’02.4″ N 5°35’30.1″ W).

Nine fungicide treatments focused on powdery mildew control were tested (Table 1): five organics (ECO, AGR, IDA, SIP, and CER), two based on integrated disease management (IDM and IDM+), and two conventional treatments based on chemical products (CON and CON +). Additionally, non-treated plants were used as a negative control (CC). Organic and integrated disease management treatments were applied with a backpack spray to cover the entire plant. Conventional treatments were applied with a trailed atomizer. The blocks corresponding to these treatments were implemented 3 rows away from the experimental yard to avoid the drift of the product. Treatments with the symbol + were applied with a diminution of 30% of the product following the recommendations of the use of Nutragreen^®^ (Cercedilla, Madrid, Spain), a nanoscale carrier that improves organic substance transport [21].

A total of 6 and 5 applications were implemented every two weeks in MR and SAFE-ULE (Table 1), respectively, except for the application of the conventional treatments (CON and CON+) in SAFE-ULE from the 3rd application, when Sulfur 80% [WP] was applied weekly. The treatments started when powdery mildew symptoms were first observed and finished two weeks before harvest. Thus, the start date of the application was 5 June 2020 in MR (phenological state among 36–38) and 19 June 2020 in SAFE-ULE (phenological stage among 38–39). The last applications were 14 August and 24 August 2020, respectively.

Both experiments were carried out in 14 rows (3 m apart) with 48 and 40 ‘Nugget’ cultivar plants (1.5 m apart) in each row in MR and SAFE-ULE, respectively. The height of the wire-work was 6 m in both locations. The number of strings per rootstock and hop bines trained to each string varied between plants due to the natural plant variability.

A randomized complete block design was conducted, with 10 treatments (9 fungicides + negative control) and 3 replicates per treatment (30 plots); conventional treatments were arranged in adjacent areas to the other treatments to avoid product drift. The experimental unit contained 10 and 12 plants in SAFE-ULE and MR, respectively. The area of the experimental unit was 45 m^2^ (10 plants × 1.5 m distance between plants × 3 m between rows) in SAFE-ULE and 54 m^2^ (12 plants × 1.5 m distance between plants × 3 m between rows) in MR.

### 2.2. Leaf Sampling

**Figure 1:**
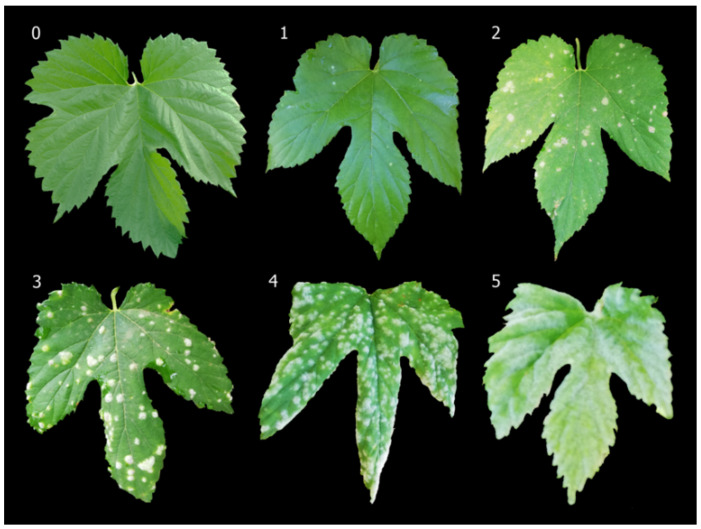
0–5 scale symptoms in leaves exerted by *Podosphaera macularis* colonization where: 0 = no visible symptoms, 1 = up to 25% of the total area colonized by *P. macularis*, 2 = 25 to 50% of the total area colonized by *P. macularis*, 3 = 50 to 75% of the total area colonized by *P. macularis*, 4 = 75 to 99% of the total area colonized by *P. macularis*, and 5 = 100% of the total area colonized by *P. macularis*.

Disease symptom in leaves, defined as *P. macularis* colonization, was scored along the bioassay using a 0 to 5 rating scale according to the area affected by *P. macularis* in leaves expressed as a percentage of the total area colonized by *P. macularis*, therefore: 0 = no visible symptoms, 1 = up to 25%, 2 = 25 to 50%, 3 = 50 to 75%, 4 = 75 to 99%, and 5 = 100% (Figure 1). In each plant, 10 leaves with the highest symptoms were selected for the evaluation. Scores were recorded weekly for 9 and 10 weeks in MR and SAFE-ULE, respectively.

Disease severity data were used to determine the following parameters for both locations: (i) disease intensity index in leaves (*DIIL*) at the end of the experiment defined as Equation (1):

(1) DIIL=(∑Si×Ni)(5×Nt)

where *Si* is the severity of the symptoms, *Ni* is the number of leaves with *Si* symptoms severity, and *Nt* the total number of leaves evaluated (10); (ii) disease incidence established as the percentage of affected plants at the end of the assay (DIL); and (iii) standardized area under the disease progress curve of DIIL plotted over time (days) (*SAUDPC*) calculated according to Equation (2) [22].

(2) $$SAUDPC=\overset{n-1}{\underset{i=1}{\sum }}\left(y_{i}+y_{i+1}\right)/2\;x\;\left(t_{i+1}-t_{i}\right)\;$$

where *n* is the number of evaluations, *y* the severity, and *t* the number of days after the first application of the treatments.

### 2.3. Cone Sampling

At the end of the season, each plant was harvested and weighted. Cones were collected using a peeling machine and fresh weighted. Two cone samples were collected per treatment and replicate.

**Figure 2:**
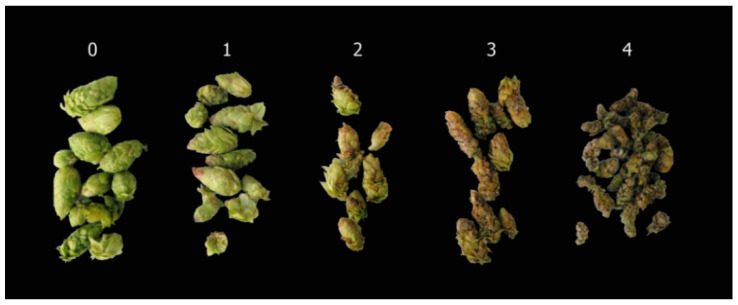
0–4 color-size scale symptoms in cones exerted by *Podosphaera macularis* colonization where: 0 = no distortion caused by *P. macularis*, 1 = distortion from *P. macularis* on up to 25% of the cone area and fully elongated cone, 2 = 25 to 50% of the cone affected by *P. macularis* colonization and cone elongated to greater than 75% of the length of an unaffected cone, 3 = 50 to 75% of the cone with distortion caused *by P. macularis* and elongated cone 50 to 75% of the length of an unaffected cone, and 4 = 75 to 100% of the cone with distortion and cones 25 to 50% of the length of an unaffected cone.

The first sample (50 g) was used to evaluate color defects and cone size reduction caused by powdery mildew infection. The evaluation followed a cone 0–4 color-size scale adapted from Twomey et al. [23]: 0 = no distortion caused by *P. macularis*, 1 = distortion from *P. macularis* on up to 25% of the cone area and fully elongated cone, 2 = 25 to 50% of the cone affected by *P. macularis* colonization and cone elongated to greater than 75% of the length of an unaffected cone, 3 = 50 to 75% of the cone with distortion caused by *P. macularis* and elongated cone 50 to 75% of the length of an unaffected cone, and 4 = 75 to 100% of the cone with distortion and cones 25 to 50% of the length of an unaffected cone (Figure 2).

The disease incidence index in cones (*DIIC*) was calculated with Equation (3):

(3) DIIC=(∑Si×Ni)(4×Nt)

where *Si* is the severity of the symptoms, *Ni* is the percentage of cones with *Si* symptom severity, and *Nt* percentage of checked cones (100%). In addition, final disease incidence (DI) was also calculated for cones (DIC).

The second sample was dried at 60 °C for 24 h until reaching a water content percentage of around 10%. Then, the biomass was ground to powder in an SM100 Comfort rotary mill (Retsch GmbH, Haan, Germany), alpha acids content was measured by Lead Conductance Value (LCV) following a modification of the method described by EBC 7.4 [24]. Briefly, the bitter substances were extracted with toluene from the freshly ground hops. Afterward, an aliquot of the toluene extract was diluted with methanol, and the LCV of the bitter substances in the resulting solution was determined by conductimetric titration with lead acetate solution using an 856 Conductivity Module (Metrohm AG, Herisau, Switzerland) with an 800 Dosino (Metrohm AG, Herisau, Switzerland).

Finally, a quality index was calculated by multiplying the yield index and the α-acid content.

### 2.4. Statistical Analysis

All parameters calculated were subjected to ANOVA using IBM SPSS Statistics for Windows Version 26.0 (IBM Corp.: Armonk, NY, USA). Treatment means were compared using Fisher’s protected least significant difference (LSD) test at α = 0.05.

Additionally, the linear correlation coefficient (*r*) was obtained for all parameter calculated using the Equation (4):

(4) $$r=\frac{\sum_{i=1}^{n}\left(x_{i}-\bar{x}\right)\left(y_{i}-\bar{y}\right)}{\sqrt{\sum_{i=1}^{n}{\left(x_{i}-\bar{x}\right)}^{2}\sum_{i=1}^{n}{\left(y_{i}-\bar{y}\right)}^{2}}}$$

where *x* and *y* are the variables for which we are interested to explore the relationship.

## 3. Results

### 3.1. Control of Podosphaera macularis Colonization in Leaves

Analysis of colonization in hop leaves in MR showed that all treatments, except for SIP, displayed the capability of controlling *P. macularis* colonization to a certain degree, showing a significant (*p* < 0.05) reduction of all parameters calculated as observed in SAUDPC values (0.25 to 0.80), as well as in DII (3.11 to 15.56) and DI (14.44 to 52.78%) in comparison to non-treated plants (CC), where values were 1.20, 31.39, and 96.11, respectively (Table 2).

Regarding SAFE-ULE, SIP showed no significant differences (*p* ≥ 0.05) in SAUDPC compared to control plants (CC) (1.71 and 1.32, respectively) and significant differences (*p* < 0.05) in DII and DI values (43.00 and 93.33, respectively) when compared to CON treatment (16.27 and 65.66, respectively) (Table 3).

In both locations, the organic treatments AGR and CER showed no significant differences to CON and CON+, based on chemical compounds, controlling *P. macularis* on leaves.

### 3.2. Control of Podosphaera macularis Colonization in Cones

In MR (Table 2), DIIC was significantly different for the treatments CON+, SIP, and IDM (29.01, 39.18, and 35.40) compared to CC (44.97), which showed no significant differences to the other organic treatments (45.06 to 46.87). For DIC, treatments CON+ and SIP presented the lowest percentage (74.27% and 83.24%), with significant differences to CC (93.30%). None of the treatments were able to reduce cone infection.

In SAFE-ULE (Table 3), the conventional treatments (CON and CON+) and IDM+ (30.62 to 33.29) showed significant differences in DIIC compared to non-treated plants (42.40), and were able to control the incidence of PM on the cones to some degree. The organic treatments did not reduce the disease incidence in cones (45.48 to 47.79), showing no significant differences to CC. DIC values were not significantly different from CC.

### 3.3. Yield Index

In MR (Table 2), CON showed the best value of yield index (0.37 kg of cone/kg of plant) with no significant differences with CON+ and IDM (0.33 to 0.34 kg of cone/kg of plant, respectively). All organic treatments showed no significant differences with CC (0.24 to 0.29 kg of cone/kg of plant). In SAFE-ULE (Table 3), all treatments showed no significant differences to CC (0.38 to 0.46 kg of cone/kg of plant).

### 3.4. α-acid Yield

Concerning MR (Table 2), all treatments, excluding ECO (6.85%), showed no significant (*p* ≥ 0.05) differences in α-acid content compared to CC. Regarding SAFE-ULE (Table 3), α-acid content in CON and CON+ treatments showed the highest value (7.79% and 7.62%) with significant differences to CC (6.84%). All organic treatments (6.63% to 6.96%) and IDM and IDM+ (6.86% and 6.79%) showed no significant differences to CC.

### 3.5. Quality Index

In MR (Table 2), the conventional treatments CON and CON+ and IDM showed significantly higher quality indexes (2.89, 2.86, and 2.78, respectively) compared to the CC treatment (2.03). IDM+ (2.25) showed no significant differences to IDM.

In SAFE-ULE (Table 3), all treatments showed no significant differences to CC (2.55 to 3.51) except for CON+, which showed the highest quality index (3.51).

### 3.6. Correlation Among Variables

Several linear correlations were observed among the parameters analyzed. In MR (Table 4), the parameters in leaves, SAUDPC, DIIL, and DIL, were positively correlated (0.98 to 0.99). Cone yield index was negatively correlated with the infection parameters in leaves, SAUDPC, DIIL, and DIL (−0.47 to −0.54), as well as the disease infection index in cones (DIIC) (−0.66). As observed in leaves, DIIC and DIC showed a positive correlation (0.73). On the other hand, α-acid content was strongly correlated with yield index (0.71), and negatively correlated with DIIC and DIC (−0.77 and −0.42, respectively).

Regarding SAFE-ULE (Table 5), a strong correlation was found between SAUDPC, DIIL, and DIL in leaves (0.80 to 0.93). As in MR, the yield index correlated negatively with DIIL, DIL (−0.75 and −0.39), and DIIC (−0.61). On the other hand, DIC showed a strong positive correlation with DIIC (0.96). Finally, α-acid content was negatively correlated with DIIL (−0.56) and DIIC and DIC (−0.74 and −0.76, respectively), while it showed a positive correlation with yield index (0.50), as previously observed in the MR location.

## 4. Discussion

Nine treatments were applied in two separate locations to evaluate their ability to control hop powdery mildew. Five treatments were organic combinations, two based on integrated disease management, and two conventional treatments applied by hop farmers (with and without nanoscale carriers). This article aims to shed light on the effect of the colonization of leaves and cones by *P. macularis* and the influence of the different treatments on the control of powdery mildew in both tissues and observe the impact in the yield index and α-acid content in cones.

Regarding the leaf control of the inoculum, the organic treatments AGR, CER, and SIP were able to control the disease to the levels of CON treatment, a conventional treatment based on chemical synthesis compounds. It suggests that in the first stages of the development of the plant, PM can be controlled by organic treatments with comparable results to chemical-based compounds. The use of potassium hydrogen carbonate has proven effective in managing PM in other crops such as pumpkin or cucumber [25,26]. The use of sulfur, widely distributed as an organic alternative, controls PM by contact action blocking the fungus development [27].

In the control of the inoculum in cones, the organic treatments do not provide a solid solution to the disease, the best strategies to control PM in cones were CON, CON+, IDM, and IDM+ treatments. The presence of chemical fungicides in the treatments provided a better solution to reduce the inoculum when cones are already infected. Metrafenone was the chemical compound selected and applied in the CON, CON+, IDM, and IDM+ treatments in MR in one of the applications. Metrafenone is a fungicide registered in 2006 to control powdery mildew in diverse crops, mainly cereals and grapevine. It shows good performance, but some evidence has been reported on the development of resistance in wheat and grapevine [28,29]. Hence, this compound must be applied sparingly.

The organic treatments showed no significant differences to CC plants in terms of yield index in both locations. Nonetheless, in SAFE-ULE, the yield index showed no significant differences between the conventional treatments and the organic. It may be since CON in SAFE-ULE was only sulfur in the last stages.

The content of α-acid in hops is expressed as a percentage of the dry weight of the cone. It is characteristic of a cultivar, but weather conditions or farmer management can alter the proportions of resins by the weight of bracts and bracteoles [30]. Alpha-acid content showed no significant differences among treatments in SAFE-ULE. In MR, CON+ showed the highest α-acid content, with no significant differences to CON and CC.

The quality index was obtained by multiplying the yield index and the α-acid content. In MR, the conventional treatments CON and CON+ and IDM showed significantly higher quality indexes than CC treatment. In SAFE-ULE, the CON+ showed the highest value of quality index without significant differences with CON. The treatments AGR, SIP, CER, and the IDM and IDM+ treatments showed no significant differences in the quality index compared to the CON treatment alone. It suggests that the quality of the hops does not differ significantly if the treatment applied to control PM is organic-based or involved some chemically synthesized compounds.

With the presented results, a possible solution to reduce the application of chemical fungicides is a combination of organic and chemical compounds. A management design with the presence of organic and biological control agents can result in a reduction of the initial inoculum source and reduce its incidence in cones, thus leading to safer hop production as the quality index of the organic-treated cones did not differ from the conventionally treated ones.

Finally, the addition of a nanoscale carrier (Nutragreen^®^) to CON and IDM treatments along with a decrease of 30% of the pesticide dose showed that treatments of CON+ and IDM+ were still efficient in controlling powdery mildew and, in the case of CON+, maintained yield levels and even enhanced the α-acid content in cones. This finding, which matches with results found in the reduction of pesticides in pear trees [21], is highly relevant when a reduction in chemical products is an urgent necessity in modern agriculture due to a lack of control in their application in the past.

Some authors previously described that *P. macularis* colonization in leaves also produced the colonization of cones [8,9,11]. However, studies revealing the link between colonization of these plant structures with yield and α-acid content are scarce. Correlations carried out among calculated parameters in our study (SAUDPC, DIIL, DIL yield index, DIIC, DIC, and α-acid content) established in two locations showed a negative correlation between both DIIL and DIIC, and yield index; as well as between DIIC and α-acid content, with the latter already having been mentioned in Gent et al. [9]. Thus, disease intensity in leaves and cones was a crucial factor in the yield index, where high disease values were accompanied by a decrease in crop yield. Moreover, the disease intensity in cones also produced a reduction of α-acid content, thus decreasing cone quality.

## 5. Conclusions

Results presented here reveal that when *P. macularis* colonizes leaves and cones, a decline in yield index is observed. In addition, higher levels of powdery mildew in cones are accompanied by a reduction in α-acid yield. Concerning the nine treatments tested, although most organic treatments can diminish *P. macularis* in leaves, no effect has been observed in cones (except for SIP treatment, for which findings were the opposite) either in yield index and α-acid yield and therefore in the quality index. On the contrary, the utilization of conventional treatments reduced infection in leaves and cones, which increased the cone quantity and quality compared to non-treated plants. Likewise, the utilization of products framed on integrated disease management provided satisfactory results, diminishing powdery mildew in both analyzed tissues, as IDM improved yield and quality indices in both locations and IDM+ in MR. Finally, the dose reduction, due to the use of the Nutragreen^®^ nano-scale carrier, does not affect the beneficial effects of the tested products. Additional experiments would be of great interest to deepen knowledge of both the use of integrated disease management and Nutragreen^®^ as strategies to reduce the use of chemical products in the control of powdery mildew by hop farmers.

## Figures and Tables

**Table 1:** Treatments, product name, active ingredient, and application timing tested in hop cultivar ‘Nugget’.

			Application Timing ^1^					
Treatment	Product Name	Active Ingredient	1 ^2^	2	3	4	5	6
**ECO**	LECTUM	Soy lecithin (NON-GMO) + *Equisetum arvense* L. extract [EC] W/W	√	√	√	√	√	√
**AGR**	ACTILEAF	Cerevisane 94,1% [WP] W/W	√	√	√	√	√	
	HELIOSOUFRE	Sulfur 72% [+ Pinolene] [SC] W/V	√	√	√	√	√	√
	VITISAN	Potassium hydrogen carbonate 99,99% [SP] W/W		√	√	√	√	√
**IDA**	NELA	Cinnamon extract [EC] W/W	√	√	√	√	√	√
	S-SYSTEM	Bioassimilable sulfur 32% + Mn + Zn [EC] W/W	√	√	√	√	√	√
**SIP**	ARAW	Eugenol 3.3%, Geraniol and Thymol 6.6% [CS] W/V	√	√	√	√	√	√
**CER**	ARMICARB	Potassium hydrogen carbonate 85% W/W	√	√	√	√	√	√
	AMYLO-X	*Bacillus amyloliquefaciens* subsp. *plantarum* (strain D747) 25% [WG] W/W				√	√	√
**IDM**	HELIOSOUFRE	Sulfur 72% [+ Pinolene] [SC] W/V	√	√	√			
	VITISAN	Potassium hydrogen carbonate 99,99% [SP] W/W	√	√	√			
	VIVANDO	Metrafenone 50% [SC] W/V				√		√ ^3^
	BELLIS	Boscalid 25.2% + Piraclostrobin 12.8% [WG] W/W					√	√ ^2^
**IDM +**	IDM + Nutragreen	IDM + Nutragreen (10^−4^ volumen dilution)						
**CON MR**	SYSTHANE FORTE	Myclobutanil 24% [EC] W/V	√	√	√			
	VIVANDO	Metrafenone 50% [SC] W/V				√		
	LUNA SENSATION	Fluopyram 25% + trifloxystrobin 25% [SC] W/V					√	
**CON MR +**	CON MR + Nutragreen	CON MR + Nutragreen (10^−4^ volumen dilution)						
**CON SAFE-ULE**	NIMROD Quattro	Bupirimate 25% [EC] W/V		√	√	√		
	Sulfur 80% [WP] ^4^	Sulfur 80% [WP] W/W					√	√
**CON SAFE-ULE +**	CON SAFE-ULE + Nutragreen	CON SAFE-ULE + Nutragreen (10^−4^ volumen dilution)						

^1^ Numbers indicate the week where the product was applied. ^2^ In MR. ^3^ In SAFE-ULE. ^4^ Weekly application. W/W: Weight/Weight. W/V: Weight/Volume. EC: Emulsifiable concentrate. SC: Suspension concentrate. WP: Wettable powder. SP: Soluble powder. CS: Capsule suspensions. WG: Water dispersible granules.

**Table 2:** Colonization ability of *Podosphaera macularis* in leaf and cone, and yield, α-acid content, and quality index in cones after treatment application at La Milla del Río (MR).

Treatment ^1^	Leaf ^2^			Cone ^3^				
	SAUDPC	DIIL	DIL (%)	DIIC	DIC (%)	Yield Index (kg cone/kg Plant)	α-acid Yield (%)	Quality Index
**CON**	0.28d	4.44c	20.83cd	39.9bcd	95.27a	0.37a	7.81abc	2.89a
**CON+**	0.52cd	7.72bc	35.28bcd	29.01e	74.27d	0.33ab	8.67a	2.86a
**CC**	1.20a	31.39a	96.11a	44.97ab	93.30ab	0.26c	7.81abc	2.03cd
**ECO**	0.80bc	15.56b	52.78b	45.06ab	86.99abc	0.24c	6.85d	1.64d
**AGR**	0.53cd	10.50bc	44.17bc	46.34a	91.13abc	0.26c	7.50bcd	1.95cd
**IDA**	0.25d	3.11c	14.44d	45.50ab	85.44bc	0.27c	7.55bcd	2.04cd
**SIP**	1.05ab	27.67a	84.17a	39.18cd	83.24cd	0.26c	7.19cd	1.87cd
**CER**	0.44d	6.61bc	28.06bcd	46.87a	92.43abc	0.29bc	7.32bcd	2.12c
**IDM**	0.52cd	10.22bc	45.56bc	35.4d	85.05bc	0.34ab	8.19ab	2.78ab
**IDM+**	0.55cd	11.72bc	51.94b	41.76abc	90.12abc	0.29bc	7.76abc	2.25bc

^1^ The code of the treatments is in Table 1. ^2^ SAUDPC: standardized area under the disease progress curve; DIIL: disease intensity index in leaves; DIL: disease incidence established as the percentage of affected plants at the end of the assay. ^3^ DIIC: disease incidence index in cones; DIC: final disease incidence in cones.

**Table 3:** Colonization ability of *Podosphaera macularis* in leaf and cone, and yield, α-acid content, and quality index in cones after treatment application at the School of Agrarian and Forest Engineering at the University of León (SAFE-ULE).

Treatment ^1^	Leaf ^2^			Cone ^3^				
	SAUDPC	DIIL	DIL (%)	DIIC	DIC (%)	Yield Index (kg cone/kg plant)	α-acid Yield (%)	Quality Index
**CON**	0.80c	16.27b	65.66bcd	33.29c	89.61b	0.45a	7.79a	3.51ab
**CON+**	0.70c	15.31b	60.42bcd	30.62c	87.53b	0.46a	7.62a	3.51a
**CC**	1.32ab	32.27ab	81.33ab	42.40ab	93.43ab	0.43abc	6.84b	2.94bcd
**ECO**	0.73c	21.4b	59.33cd	45.48ab	95.67ab	0.39bc	6.96b	2.71cd
**AGR**	0.71c	20.93b	61.33bcd	47.13a	97.48a	0.44ab	6.65b	2.93bcd
**IDA**	0.87bc	35.53ab	79.67abc	46.30ab	96.98ab	0.38c	6.70b	2.55d
**SIP**	1.71a	43.00a	93.33a	47.11a	95.76ab	0.42abc	6.63b	2.78bcd
**CER**	0.72c	21.13b	65.66bcd	47.79a	96.38ab	0.43abc	6.75b	2.90bcd
**IDM**	0.83bc	17.13b	51.67d	38.38bc	94.60ab	0.45a	6.86b	3.09abc
**IDM+**	1.02bc	19.20b	62.33bcd	32.77c	89.20b	0.43abc	6.79b	2.92bcd

^1^ The code of the treatments is in Table 1. ^2^ SAUDPC: standardized area under the disease progress curve; DIIL: disease intensity index in leaves; DIL: disease incidence established as the percentage of affected plants at the end of the assay. ^3^ DIIC: disease incidence index in cones; DIC: final disease incidence in cones.

**Table 4:** Linear correlation coefficient among parameters calculated for La Milla del Río (MR) location.

		Leaf ^1^			Cone ^2^			
		SAUDPC	DIIL	DIL (%)	Yield Index (kg cone/kg Plant)	DIIC	DIC (%)	α-acid Content (%)
**Leaf ^1^**	**SAUDPC**	1	0.99	0.98	−0.54	0.07	−0.01	−0.25
	**DIIL**		1	0.99	−0.50	0.10	0.06	−0.24
	**DIL (%)**			1	−0.47	0.04	0.04	−0.17
**Cone ^2^**	**Yield Index (kg cone/kg plant)**				1	−0.66	−0.09	0.71
	**DIIC**					1	0.73	−0.77
	**DIC (%)**						1	−0.42
	**α-acid content (%)**							1

^1^ SAUDPC: standardized area under the disease progress curve; DIIL: disease intensity index in leaves; DIL: disease incidence established as the percentage of affected plants at the end of the assay. ^2^ DIIC: disease incidence index in cones; DIC: final disease incidence in cones.

**Table 5:** Linear correlation coefficient among parameters calculated for SAFE-ULE location.

		Leaf ^1^			Cone ^2^			
		SAUDPC	DIIL	DIL (%)	Yield Index (kg cone/kg Plant)	DIIC	DIC (%)	α-acid Content (%)
**Leaf ^1^**	**SAUDPC**	1	0.80	0.83	−0.13	0.21	0.09	−0.36
	**DIIL**		1	0.93	−0.75	0.60	0.50	−0.56
	**DIL (%)**			1	−0.39	0.42	0.26	−0.32
**Cone ^2^**	**Yield Index (kg cone/kg plant)**				1	−0.61	−0.57	0.50
	**DIIC**					1	0.96	−0.74
	**DIC (%)**						1	−0.76
	**α-acid content (%)**							1

^1^ SAUDPC: standardized area under the disease progress curve; DIIL: disease intensity index in leaves; DIL: disease incidence established as the percentage of affected plants at the end of the assay. ^2^ DIIC: disease incidence index in cones; DIC: final disease incidence in cones.

## Data Availability

The data presented in this study are available on request from the corresponding author.
